# Isothiocyanates attenuate heparin‐induced proliferation of colon cancer cells in vitro

**DOI:** 10.1002/fsn3.4296

**Published:** 2024-08-13

**Authors:** Yizi Zhang, Karabi Saha, Raj Nandani, Jiahui Yuan, Moul Dey, Zhengrong Gu

**Affiliations:** ^1^ Department of Agricultural and Biosystems Engineering South Dakota State University Brookings South Dakota USA; ^2^ Department of Pharmaceutical Sciences South Dakota State University Brookings South Dakota USA; ^3^ School of Health and Consumer Sciences South Dakota State University Brookings South Dakota USA

**Keywords:** apoptosis, colon cancer, Erb‐B family, isothiocyanates, low‐molecular‐weight heparin

## Abstract

Isothiocyanates (ITCs), prevalent in cruciferous vegetables, are known for their anticarcinogenic properties. Prior research has indicated that heparin can stimulate the growth of colon cancer cells. However, the implications of ITCs in the diet of cancer patients receiving heparin‐based therapies have yet to be fully understood. This exploratory in vitro study examines the proliferative effects of low‐molecular‐weight heparin (LMWH) on human colon cancer cells and assesses the antiproliferative potential of four ITC compounds, exploring possible epidermal growth factor family of receptor tyrosine kinases (Erb‐B) related mechanisms. We evaluated cell viability in HCT‐116 and HT‐29 cell lines following treatment with ITCs alone or combined with LMWH (20 μg/mL) at various concentrations (1–100 μM). Clonogenic and wound‐healing assays were performed after 24 h of treatment with 5 μM ITCs. Additionally, messenger RNA (mRNA) and protein expression of Erb‐B family genes was measured using quantitative polymerase chain reaction (qPCR) and Western blotting. Statistical analysis was conducted using analysis of variance (ANOVA) with Dunnett's post hoc test. Results indicated that the half‐maximal inhibitory concentration (IC_50_) values for Phenylethyl isothiocyanate (PEITC), Benzyl isothiocyanate (BITC), and Sulforaphane (SFN) were lower than those of Allyl isothiocyanate (AITC) in LMWH‐stimulated HCT‐116 (20.77, 19.10, and 44.05 μM, respectively) and HT‐29 (74.94, 26.77, and 43.49 μM, respectively). PEITC and SFN significantly reduced ErbB1 (epidermal growth factor receptor (EGFR)) and ErbB4 (receptor tyrosine‐protein kinase erbB‐4) expression, while BITC decreased ErbB2 (receptor tyrosine‐protein kinase erbB‐2) and transforming growth factor beta (TGF‐*β*) expression in HCT‐116 cells (all, *p* < .05). PEITC, BITC, and SFN also increased proapoptotic Bax expression and decreased the antiapoptotic B‐cell lymphoma 2 (Bcl‐2) expression (all, *p* < .05). These findings suggest that specific ITCs may mitigate cancer cell proliferation induced by LMWH in cancer therapies, highlighting their potential therapeutic efficacy.

## Graphical abstract

Low‐molecular‐weight heparin (LMHW) stimulates the growth of colon cancer cells. Isothiocyanates (ITCs) can inhibit the proliferation of colon cancer cells induced by heparin 3. The mechanism might contribute to the downregulation of epidermal growth factor family of receptor tyrosine kinases (Erb‐B) family protein and messenger RNA (mRNA) expression, transforming growth factor beta (TGF‐*β*), and regulatory apoptotic factors.

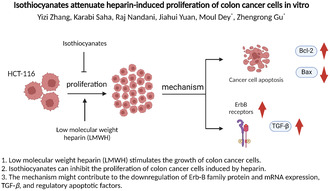

## 1 INTRODUCTION

Within the realm of organic chemistry, isothiocyanates (ITCs) represent the functional group −N=C=S, formed by replacing the oxygen in the isocyanate group with sulfur (Zhang & Talalay, 1994). ITCs, present as glycosylates, are abundant in cruciferous vegetables like broccoli, cabbage, cauliflower, and kale, with especially high concentrations found in broccoli sprouts (Vanduchova et al., 2019). They largely contribute to the distinctive flavor of these vegetables. Common ITCs used in cancer research include Phenylethyl Isothiocyanate (PEITC) (Liu et al., 2013), Benzyl isothiocyanate (BITC) (Ding et al., 2021), Sulforaphane (SFN) (Khan et al., 2022), and Allyl isothiocyanate (AITC) (Chang et al., 2021). Epidemiological evidence suggests that consuming cruciferous vegetables reduces cancer incidence. Naturally occurring ITCs, product of glycosylate hydrolysis, have attracted significant interest for their established antitumor properties (Cedrowski et al., 2021). Numerous in vitro and in vivo studies have shown that ITCs inhibit various cancer cells and may serve as potential cancer therapeutic agents (Dinh et al., 2021; Soundararajan & Kim, 2018). Recently, Jing et al. found that isothiocyanate from *Moringa oleifera* seeds inhibits the growth and migration of renal cancer cells by regulating the protein tyrosine phosphatase‐1B (PTP1B)‐dependent proto‐oncogene tyrosine‐protein kinase (Src)/rat sarcoma virus (Ras)/rapidly accelerated fibrosarcoma (Raf)/extracellular signal‐regulated kinase (ERK) signaling pathway (Xie et al., 2021). Dietary BITC administration inhibits the development of estrogen receptor‐negative breast cancer in mouse mammary tumor virus (MMTV)‐neu transgenic mice (Roy et al., 2019). SFN has been shown to suppress breast cancer cell proliferation and exhibit anticancer properties in gastrointestinal cancers (Cao et al., 2018; Mondal et al., 2016). Nevertheless, further exploration is required to fully understand the anticancer mechanisms of ITCs and potential drug interactions.

Heparin, a natural anticoagulant primarily derived from pig intestines, is commonly used in cancer patients to prevent blood clots due to their increased risk of Venous Thromboembolism (VTE). Although effective at reducing VTE incidence, the use of heparin in cancer patients necessitates further research to thoroughly assess any potential adverse effects (Fagarasanu et al., 2016; Guo et al., 2017; Rasmussen et al., 2009). As a highly sulfated glycosaminoglycan, various studies indicate that different forms of heparin, such as unfractionated heparin, low‐molecular‐weight heparin (LMWH), and heparin derivatives, can exacerbate cancer cell proliferation, adhesion, angiogenesis, migration, and invasion through diverse mechanisms (Afratis et al., 2017; Ma et al., 2020). LMWH, obtained from ordinary heparin through specific enzymatic hydrolysis, features a much smaller molecular weight. This characteristic leads to a more uniform molecular size and distribution, playing a crucial role in anticoagulant therapy (Malloy et al., 2018).

Notably, the organ‐specific extracellular matrix plays a critical role in influencing metastasis development by modulating tumor cell proliferation. Experimental evidence suggests that the composition of the surrounding extracellular matrix can determine the effects of exogenous glycosamines on various cellular functions to a certain extent (Chatzinikolaou et al., 2008). The extracellular matrix derived from hepatocellular sources has been observed to promote the proliferation of colon cancer cell lines through the upregulation of Erb‐B family tyrosine kinase receptors (Fishman et al., 2002; Zvibel et al., 2001). Additionally, exogenous heparin has been found to stimulate colon cancer cell proliferation. Treatment of HT‐29 and SW1116 cells with heparin concentrations of 10 μg/mL and above resulted in significant dose‐dependent stimulation of their growth rates (Chatzinikolaou et al., 2008). Despite this, the influence of heparin on the proliferation of colon cancer cells and its underlying mechanisms remains underexplored. In this study, we hypothesize that ITCs can inhibit LMWH‐induced cancer cell proliferation. Our findings may have dietary recommendations for cancer patients undergoing heparin‐based treatments. To validate our hypothesis, we first sought to determine the effects of heparin on human cancer cell proliferation in vitro in a concentration‐dependent manner. Subsequently, we assessed the ability of ITCs to mitigate these effects. Additionally, we aimed to elucidate the potential molecular mechanisms underlying any observed inhibitory effects.

## 2 MATERIALS AND METHODS

### 2.1 Cell culture

The HCT‐116 (CCL‐247) and HT‐29 (HTB‐38) cell lines were sourced from the American Type Culture Collection (ATCC)–The Global Bioresource Center (Manassas, VA, USA). These cell lines were cultured in McCoy's 5A medium, enriched with 10% heat‐inactivated fetal bovine serum (FBS) and 1% penicillin–streptomycin. The cultures were maintained at 37°C under 5% carbon dioxide (CO_2_) atmosphere, following ATCC's guidelines. All cell culture reagents used were obtained from ATCC, unless specified otherwise.

### 2.2 IC_50_ determination

To determine the half‐maximal inhibitory concentration (IC_50_) of each sample on HCT‐116 and HT‐29 cells, concentration‐dependent cytotoxicity assays were performed. Cells were seeded in 96‐well plates at a density of 1 × 10^5^ cells/mL and treated with various concentrations of LMWH (CAS: 9005‐49‐6, MilliporeSigma™, USA) and ITC compounds—PEITC (CAS: 2257‐09‐2, Thermo Scientific Chemicals, USA), BITC (CAS: 622‐78‐6, Thermo Scientific Chemicals, USA), SFN (CAS: 4478‐93‐7, MilliporeSigma™, USA), and AITC (CAS: 57‐06‐7, Thermo Scientific Chemicals, USA) for 24 h. Subsequently, 20 mL of 3‐(4,5‐dimethylthiazol‐2‐yl)‐2,5‐diphenyltetrazolium bromide (MTT) solution (5 mg/mL, CAS: 298‐93‐1, Thermo Scientific Chemicals, USA) was added to each well. After an additional 4 h of incubation, the reaction was stopped by introducing 100 μL of dimethyl sulfoxide (DMSO) (CAS: 67‐68‐5, Thermo Scientific Chemicals, USA) per well. The optical density at 570 nm was measured using a microplate reader (Promega, USA). Cell survival percentage was calculated by comparing the absorbance of the treated cells to that of an untreated control. The viability versus concentration data were plotted, and the IC_50_ values for each compound were estimated using the logarithmic line of best‐fit formula.

### 2.3 Clonogenic assay

Clonogenic assays were performed with minor modifications, as outlined by Zhou et al. (2018). Cells were seeded at a density of 500 cells per 10‐cm plate and allowed to grow for 48 h. Afterward, the specified samples were added at a concentration of 5 μM. The spent media were replaced with fresh media containing the respective compounds every 3 days. After a 21‐day incubation period, cells were fixed with 100% methanol for 20 min and stained with 0.5% crystal violet in 25% methanol. Colonies were then photographed using a microscope (BZ‐X810, Keyence, USA) and quantified using ImageJ software (java8).

### 2.4 Wound‐healing assay

The wound‐healing assay was conducted to evaluate cell migration. Initially, 1.5 × 10^5^ cells were seeded in each well of a 6‐well plate, with 2 mL of culture medium, and incubated at 37°C in a 5% CO_2_ atmosphere until they reached complete confluence, typically within 24 h. A 20 μL pipette tip was then used to create three parallel scratches in the bottom of each well, ensuring a consistent wound width of approximately 0.5 mm. After scratching, the media were removed and the cells were washed two to three times with sterile phosphate‐buffered saline (PBS). The cells were then treated with 5 μM of either PEITC, BITC, SFN, with or without LMWH for 24 h. Images were subsequently captured using a microscope (BZ‐X810, Keyence, USA), and quantification was performed using ImageJ software (java8).

### 2.5 RNA isolation and quantitative reverse transcription polymerase chain reaction (RT‐PCR)

Cells were subjected to total RNA isolation using TRlzol Reagent (Sigma‐Aldrich, USA), followed by DNA removal using the DNA‐free DNA Removal Kit (Invitrogen, Thermo Fisher Scientific, USA). Reverse transcription was then performed using the High‐Capacity complementary DNA (cDNA) Reverse Transcription Kit (Applied Biosystems, Waltham, MA, USA). Primers, synthesized by Jerui Bioengineering Co., LTD (Shanghai, China), with their sequences provided in Table S1, were used for amplification. Quantitative PCR (qPCR) was executed using the PowerUp™ SYBR Green Master Mix (Applied Biosystems™, USA) on the Real‐Time PCR System (Applied Biosystems™, USA). Relative gene expression changes were calculated using the delta–delta threshold cycle (2−^ΔΔCt^) method and normalized to glyceraldehyde 3 phosphate dehydrogenase (GAPDH) level.

### 2.6 Western blotting

Proteins were extracted from cells using lysis buffer and centrifuged at 13,200 rpm (revolutions per minute) at 4°C. Protein concentration was quantified using a bicinchoninic acid (BCA) kit (Thermo Scientific™, USA). Each sample, containing an equal amount of protein, was boiled in sodium dodecyl sulfate‐polyacrylamide gel electrophoresis (SDS‐PAGE) loading buffer (4 × with dithiothreitol (DTT)) for 5 min. Subsequently, 20 μL of each sample was loaded for SDS‐PAGE. Protein bands were then transferred to polyvinylidene fluoride (PVDF) membranes (Millipore, USA) and blocked with 5% nonfat milk in TBST (Tris‐buffered saline containing 0.1% Tween 20) at room temperature for 2 h. The membranes were incubated overnight with primary antibodies at 4°C targeting TGF‐*β* (CST, 3709S), EGFR (ErbB1) (epidermal growth factor receptor) (CST, 4267T), HER2 (ErbB2) (receptor tyrosine‐protein kinase erbB‐2) (CST, 4290T), HER3 (ErbB3) (receptor tyrosine‐protein kinase erbB‐3) (CST, 12708T), HER4 (ErbB4) (receptor tyrosine‐protein kinase erbB‐4) (CST, 4795T), Bcl‐2 (B‐cell lymphoma 2) (CST, 3498T), Bax (Bcl‐2‐associated X protein) (CST, 2772T), and *β*‐actin (CST, 8457T). After washing with TBST (5 min each) for three times, the membranes were incubated with the secondary antibody for 50 min. The protein bands were developed in the dark for 2 min and imaged using a chemiluminescence gel imaging system (Bio‐Rad Laboratories, USA). The gray intensities of the bands were analyzed using ImageJ software (java8).

### 2.7 Statistical analysis

All data are presented as the mean ± SD, derived from a minimum of three independent experiments conducted on different days. The normality of data distribution was assessed using the Shapiro–Wilk normality test. Parametric tests were applied when the data followed a normal distribution. A comparison of multiple group means was performed using one‐way analysis of variance (ANOVA) followed by Dunnett's test, executed in GraphPad Prism 8.0 for Windows (GraphPad Software Inc., San Diego, CA, USA). For data exhibiting non‐normal distribution, the Kruskal–Wallis test was employed. A *p*‐value <0.05 was considered statistically significant for differences between groups.

## 3 RESULTS

### 3.1 Treatment effects on colon cancer cell proliferation

**FIGURE 1:**
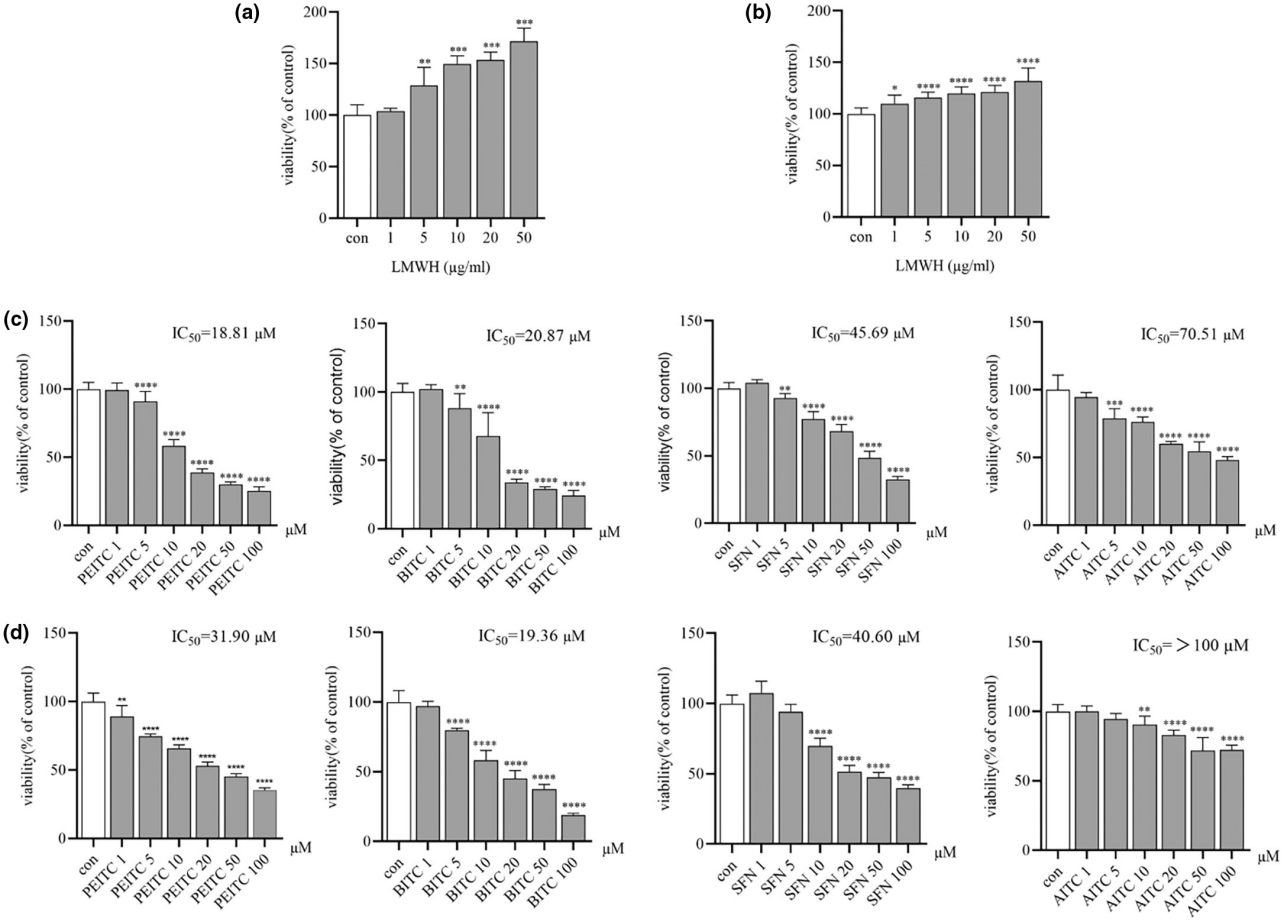
Impact of LMWH and ITCs on the viability of HCT‐116 and HT‐29 cells after a 24 h incubation. (a) LMWH‐treated HCT‐116 cell viability. (b) LMWH‐treated HT‐29 cell viability. (c) Viability of HCT‐116 cells treated with PEITC, BITC, SFN, and AITC. (d) Viability of HT‐29 cells treated with PEITC, BITC, SFN, and AITC. For A–D control group represents no treatment. Data are presented as mean ± SD (*n* ≥ 3). *p < .1, **p < .01, ***p < .001, and ****p < .0001 compared with the control group.

**FIGURE 2:**
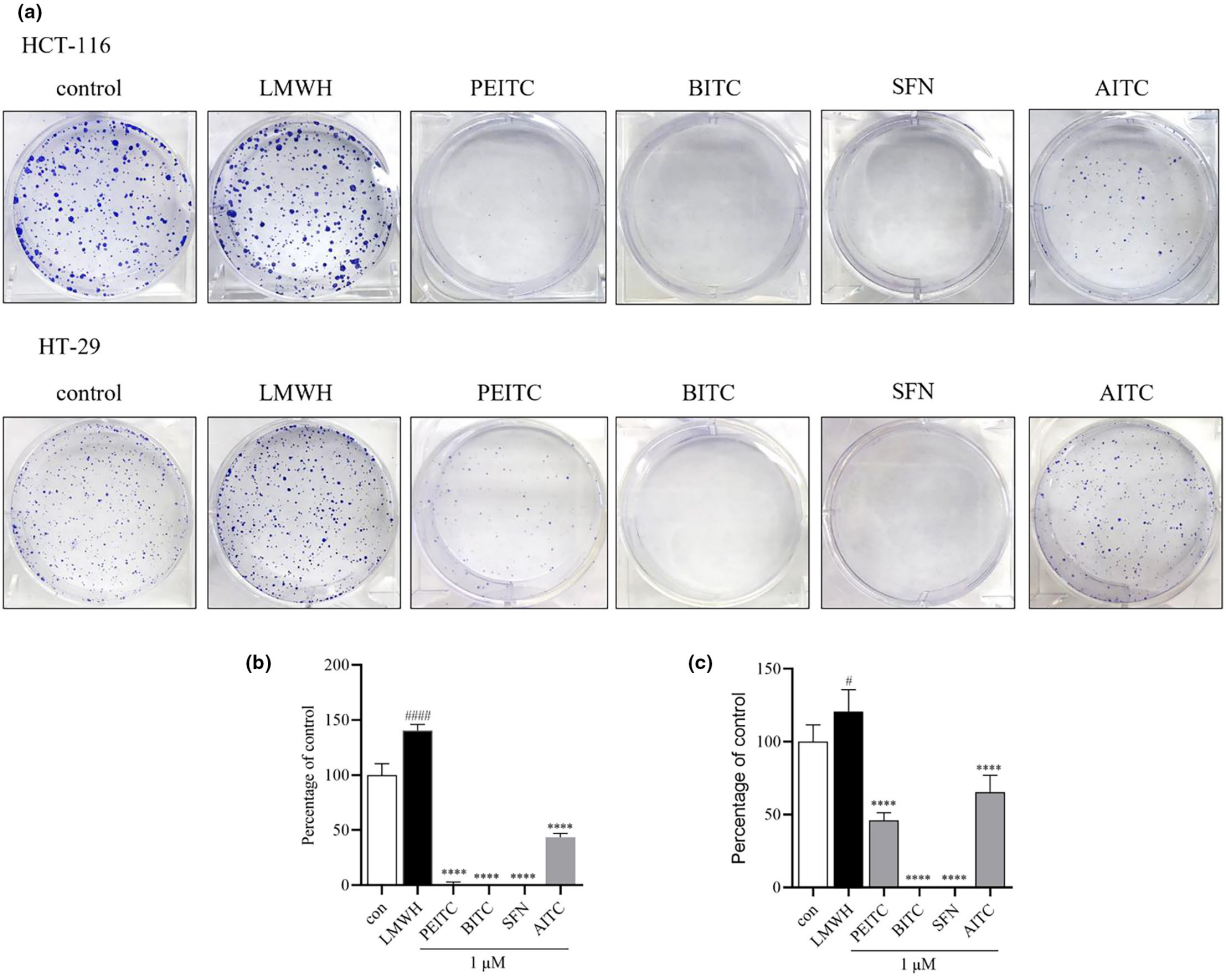
The impact of Isothiocyanates on colony formation in HCT‐116 and HT‐29 cells, respectively. (a) Representative images depicting the colony formation assay in HCT‐116 and HT‐29. (b) Quantification of counted colonies for HCT‐116 data. (c) Quantification of counted colonies for HT‐29 data. Data are presented as mean ± SD (*n* ≥ 3). ^#^ *p* < .1 and ^####^ *p* < .0001 compared with the control group, ****p < .0001 compared with the heparin group.

The study investigated the proliferative effects of varying concentrations of heparin and four isothiocyanates (ITCs)—PEITC, BITC, SFN, and AITC on two colorectal cancer cell lines, HCT‐116 and HT‐29. Heparin, at concentrations ranging from 1 to 50 μg/mL, was found to stimulate the proliferation of both cell lines, with a more pronounced effect observed in HCT‐116 cells (Figure 1a,b). Various concentrations (1–100 μM) (Du et al., 2023; Huang et al., 2018; Lu et al., 2022; Lv et al., 2020) of the ITCs (PEITC, BITC, SFN, and AITC) demonstrated varying degrees of inhibition on cell proliferation, indicating specific effects on colon cancer cell growth (Figure 1c,d). Among the tested samples, PEITC and BITC showed the most potent antiproliferative activity, with IC_50_ values of 18.81 and 31.90 μM for HCT‐116 cells, and 20.87 and 19.36 μM for HT‐29 cells, respectively. These results are consistent with those of prior studies (Chatzinikolaou et al., 2008; Cuellar‐Núñez et al., 2020; Lai et al., 2023), highlighting a stronger anticancer efficacy in HCT‐116 cells compared to HT‐29 cells. Further examination of the impact of ITCs on colony formation reveals that while LMWH enhances clonal growth, PEITC, BITC, SFN, and AITC significantly hindered it (Figure 2). Among these, PEITC, BITC, and SFN were found to be particularly effective in both proliferation and clonogenic assays, and were therefore selected for deeper investigation into their anticancer properties.

**FIGURE 3:**
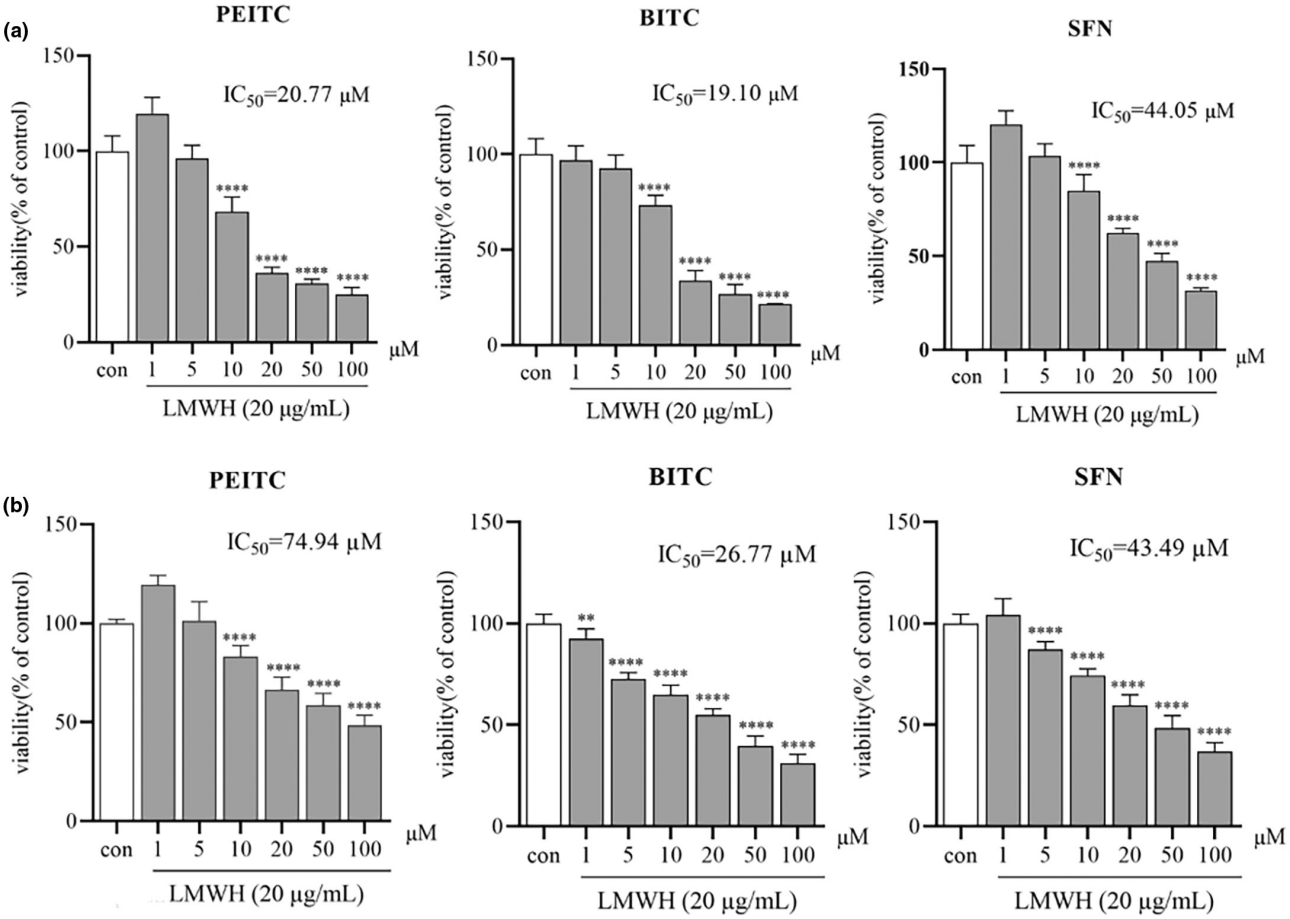
The impact of Isothiocyanates on the viability of HCT‐116 and HT‐29 cells treated with LMWH. (a) Viability of HCT‐116 cells treated with LMWH and PEITC, BITC, and SFN. (b) Viability of HT‐29 cells treated with LMWH and PEITC, BITC, and SFN. Data are presented as mean ± SD (*n* ≥ 3). **p < .01 and ****p < .0001 compared with the control group.

Subsequently, to examine whether three of ITCs (PEITC, BITC, and SFN) can inhibit the proliferation of colon cancer cells induced by LMWH (20 μg/mL), their proliferation activity was assessed at various concentrations in HCT‐116 cells. As illustrated in Figure 3, the IC_50_ values for these ITCs in HCT‐116 cells were lower, specifically 20.77 μM for PEITC, 19.10 μM for BITC, and 44.05 μM for SFN, when compared to their effects on HT‐29 cells, which were 74.94, 26.77, and 43.49 μM, respectively. These results indicate that PEITC, BITC, and SFN exhibit stronger anticancer effects in LMWH‐induced HCT‐116 cells.

### 3.2 Inhibition of colon cancer cell migration

**FIGURE 4:**
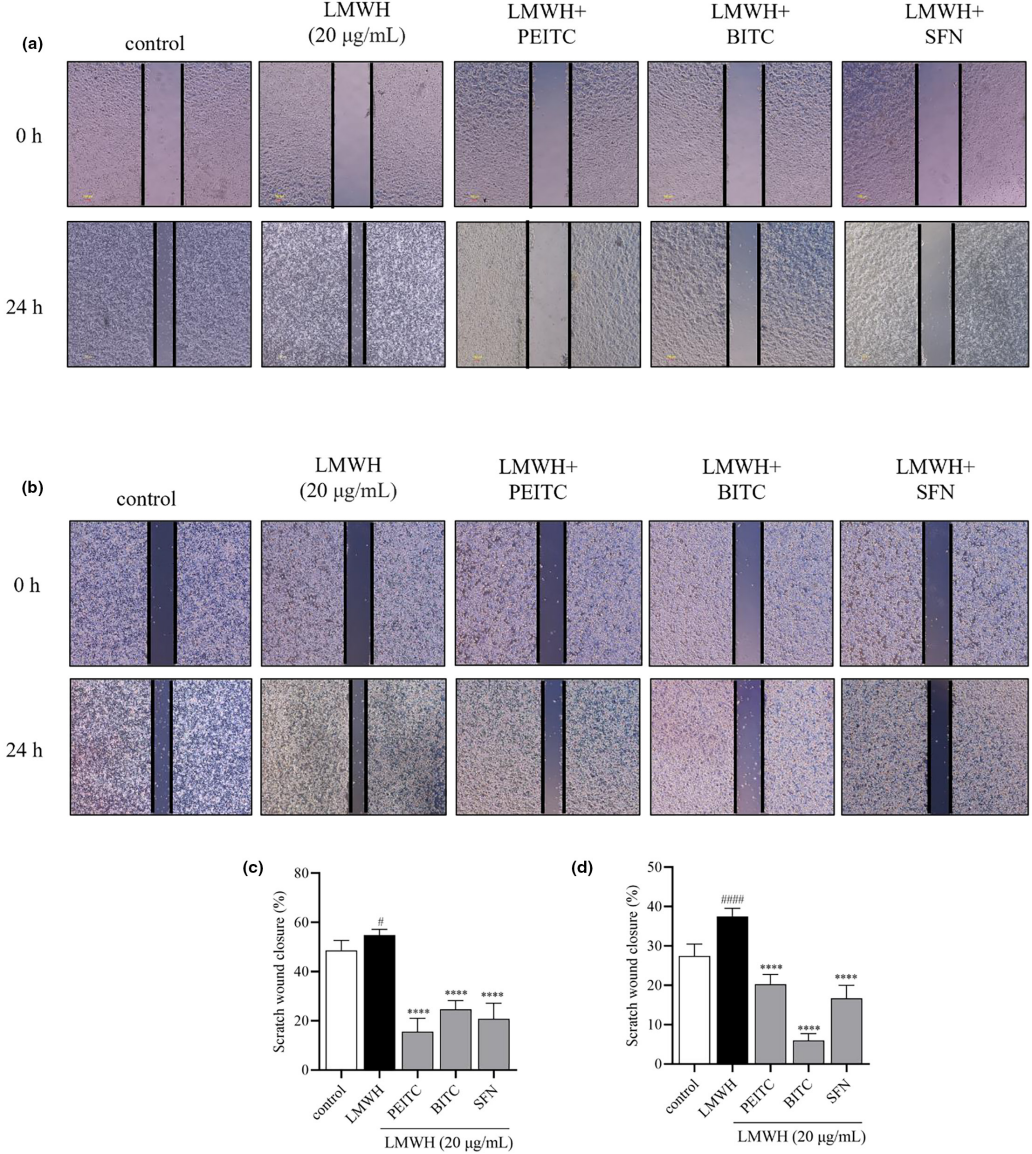
Illustrative images from the wound‐healing assay for (a) HCT‐116 cells and (b) HT‐29 cells. Quantification of the wound‐healing assay for (c) HCT‐116 cells and (d) HT‐29 cells. Data are presented as mean ± SD (*n* ≥ 3). ^#^ *p* < .1 and ^####^ *p* < .0001 compared with the control group, ****p < .0001 compared with the LMWH group.

Cell migration is a critical characteristic of invasive cancers. To investigate the antimigration effects of PEITC, BITC, and SFN induced by LMWH, wound‐healing assays were conducted on HCT‐116 cells to evaluate their capacity to inhibit cell migration. Figure 4 displays images from the assays at 0 and 24 h. While untreated cells effectively migrated to close the initially scraped area within 24 h, treatment with PEITC, BITC, and SFN (5 μM) significantly hindered cell migration in both HCT‐116 and HT‐29 cells. Due to these notable results, the HCT‐116 cell line was selected for further detailed investigations.

### 3.3 Effects of isothiocyanates on genes and proteins from the Erb‐B family, and transforming growth factor beta (TGF‐*β*) pathway

**FIGURE 5:**
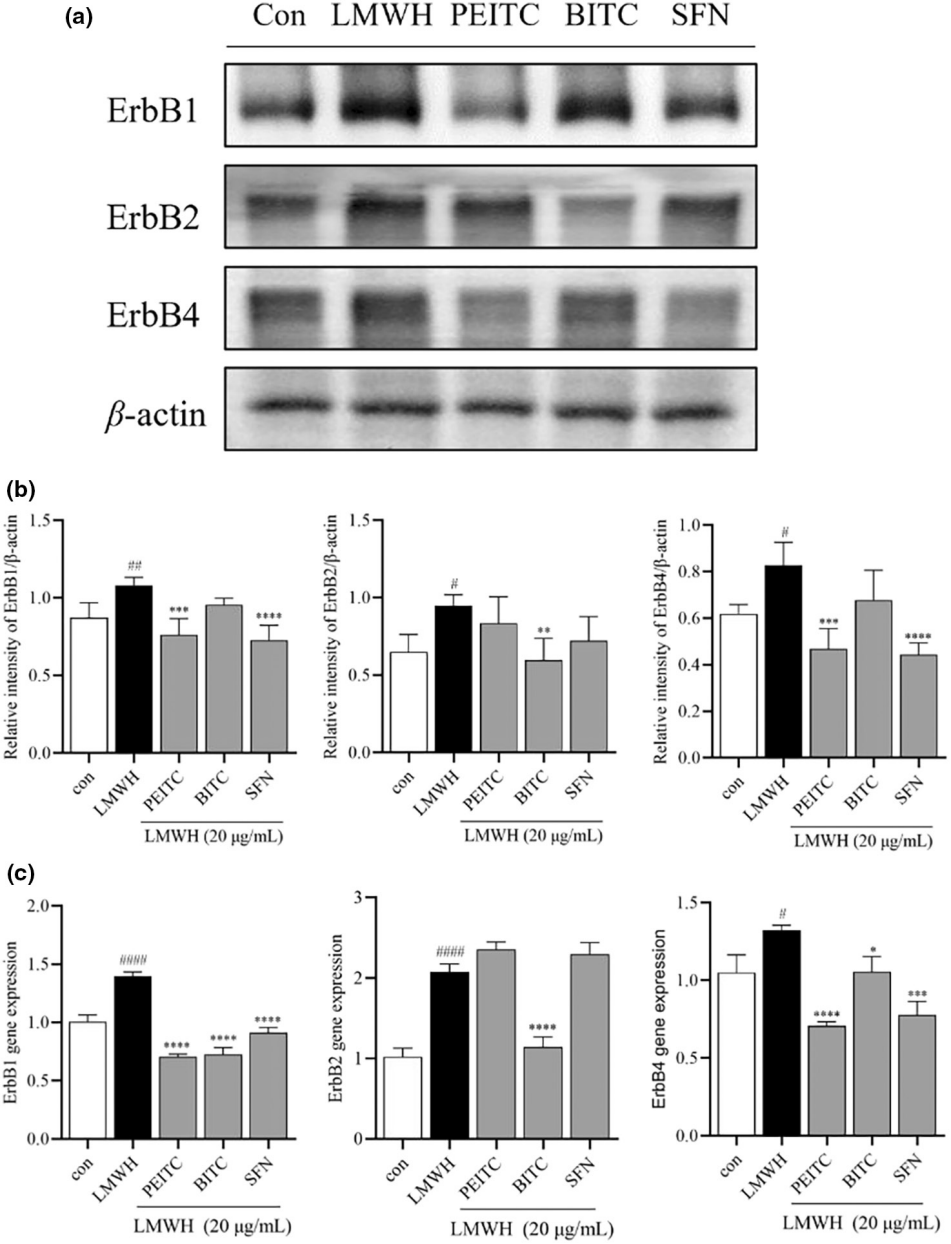
(a) Illustrative images depicting the expression levels of ErbB1, ErbB2, ErbB4, and *β*‐actin. (b) Levels of ErbB1, ErbB2, and ErbB4 normalized to *β*‐actin. (c) Gene expression levels of ErbB1, ErbB2, and ErbB4 measured by qPCR. Data are presented as mean ± SD (*n* ≥ 3). ^#^ *p* < .1, ^##^ *p* < .1, and ^####^ *p* < .0001 compared with the control group, *p < .1, **p < .01, ***p < .001, and ****p < .0001 compared with the heparin group.

**FIGURE 6:**
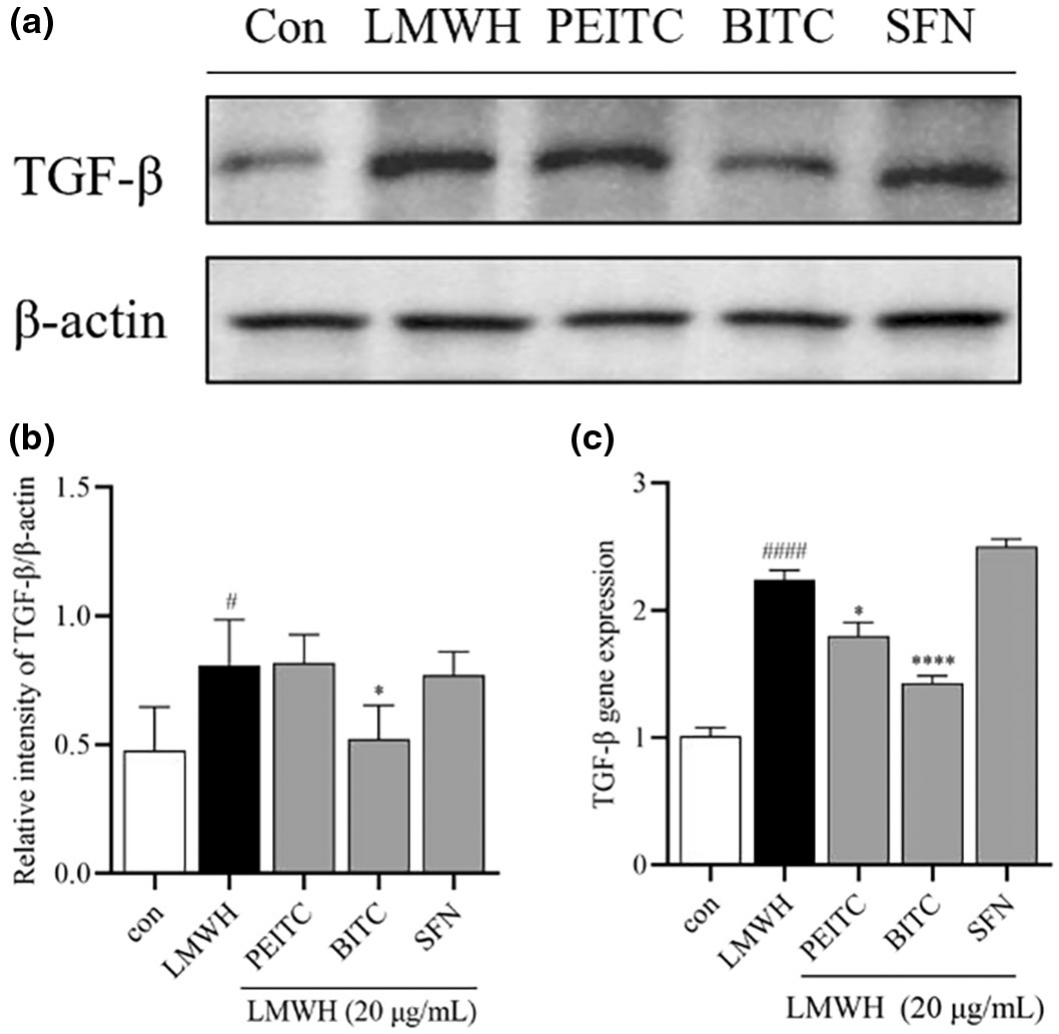
(a) Illustrative images displaying the expression levels of TGF‐*β* and *β*‐actin. (b) Normalized levels of TGF‐*β* to *β*‐actin. (c) Gene expression levels of the TGF‐*β* measured by qPCR. Data are presented as mean ± SD (*n* ≥ 3). ^#^ *p* < .1 and ^####^ *p* < .0001 compared with the control group, *p < .1 and ****p < .0001 compared with the heparin group.

It has been suggested that inhibiting the TGF‐*β*/ErbB2 (also known as HER2) pathway may reduce the progression and metastasis of certain cancers (Yingling et al., 2004). To explore the potential involvement of the Erb‐B family signaling pathway in the in vitro anticancer effects of ITCs, Western blot analysis and qPCR were used to assess the protein and gene expression of the Erb‐B family. Treatment with LMWH increased the protein and mRNA expression of ErbB1, ErbB2, and ErbB4, aligning with findings from Fishman et al. (2002). Following 24‐h treatment with 5 μM PEITC, BITC, or SFN in LMWH‐induced HCT‐116 cells, PEITC and SFN notably reduced the protein expression of ErbB1 and ErbB4, while BITC significantly decreased the protein expression of ErbB2 (Figure 5a,b). Additionally, all three ITCs significantly downregulated the mRNA expression of ErbB1 and ErbB4, with BITC also reversing the LMWH‐induced elevation in ErbB2 expression (Figure 5b). Figure 6 shows that both Western blot and qPCR results indicated an increase in TGF‐*β* protein and mRNA expression after LMWH treatment, with BITC being the only ITC to reverse this change. No significant changes were observed in the expression of ErbB3 (data not shown).

### 3.4 Effect of isothiocyanates on apoptosis‐related genes and proteins

**FIGURE 7:**
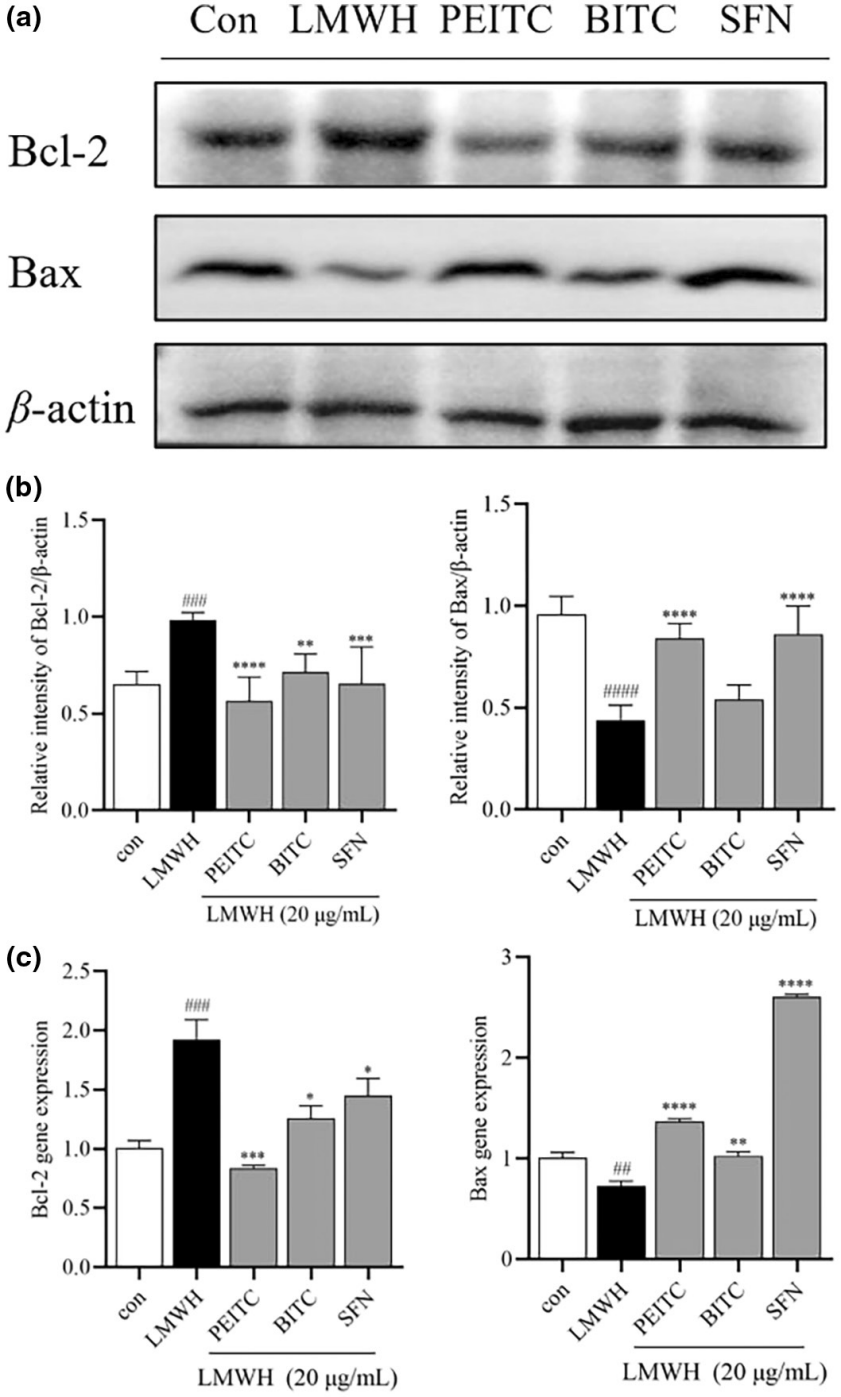
(a) Illustrative images depicting the expression levels of Bcl‐2, Bax, and *β*‐Actin. (b) Normalized levels of Bcl‐2 and Bax to *β*‐actin. (c) Gene expression levels of the Bcl‐2 and Bax measured by qPCR. Data are presented as mean ± SD (*n* ≥ 3). ^##^ *p* < .01 and ^###^ *p* < .001 compared with the control group, *p < .1, **p < .01, ***p < .001, and ****p < .0001 compared with the heparin group. ^####^ *p* < .0001.

The absence of apoptotic control is a key factor in the survival of cancer cells. Heparin treatment inhibited apoptosis in colon cancer cells, as evidenced by an increase in the antiapoptotic protein Bcl‐2 and a simultaneous decrease in the proapoptotic protein Bax. While BITC did not lead to an increase in Bax protein expression, PEITC, BITC, and SFN countered the apoptotic changes by inhibiting Bcl‐2 (Figure 7). Notably, PEITC and SFN also enhanced Bax levels, effectively promoting apoptotic mechanisms.

## 4 DISCUSSION

Cancer remains the second leading cause of death globally, with increasing incidences of lung, colorectal, breast, and prostate cancers each year (Xia et al., 2022). The challenges of high toxicity, formidable chemotherapy resistance, and negative side effects with cancer treatment drugs underscore the need for novel small molecules. These should bypass chemotherapy resistance, feature favorable toxicity profiles, and possess scientifically validated mechanisms of action, ultimately enhancing the quality of life and survival rates of patients (Zugazagoitia et al., 2016). Cruciferous vegetables contain glucosides that require enzymatic conversion by the plant enzyme myrosinase into ITCs, organic sulfur compounds with health‐promoting properties (Wang & Bao, 2021). Sulforaphane (SFN), a widely studied ITC first identified in broccoli in 1992 (Zhang et al., 1992), has demonstrated potential to prevent the development and progression of prostate cancer (Zhang et al., 2020). ITCs also exhibit synergistic effects with specific anticancer drugs, enhancing the efficacy of treatments, such as irinotecan in colorectal cancer cells (Lai et al., 2023) and cisplatin in gastric cancer (Rabben et al., 2021). Despite the recognized anticancer properties of ITCs, their clinical application has been limited by factors, such as low solubility, poor bioavailability, and instability. Recent advancements in nanoscale delivery systems have improved their stability and bioavailability, enhancing their therapeutic potential (Rizwan & Masoodi, 2023). As a result, ITCs are regarded as a promising alternative medicine candidates for cancer treatment in comparison to synthetic anticancer drugs.

There is a well‐documented relationship between cancer and thrombosis, the risk of VTE in cancer patients is 9‐fold higher than in the general population (Mulder et al., 2021). The standard preventive care includes the routine use of LMWH to mitigate potential blood clot formation. While primarily used for its antithrombotic properties, several preclinical and clinical investigations have indicated that LMWHs might also enhance the survival rates of cancer patients through mechanisms distinct from their antithrombotic properties (Kuderer et al., 2007; Ma et al., 2020). However, the precise effects of LMWH on cancer cell proliferation remain inadequately understood, with some studies indicating a potential to promote cancer cell growth (Chatzinikolaou et al., 2008; Lean et al., 2014). Our study confirms that LMWH can indeed stimulate the growth of colorectal cancer cells HCT‐116 and HT‐29, necessitating further investigations to understand this mechanism and to develop strategies to mitigate these effects. Furthermore, our research explores the anticancer effects and mechanisms of four commonly occurring isothiocyanates (PEITC, BITC, SFN, and AITC) in LMWH‐stimulated HCT‐116 cells. We observed that all four ITCs could inhibit the proliferation and clonal formation of HCT‐116 and HT‐29 cancer cells induced by heparin, with PEITC, BITC, and SFN showing superior effects. Additionally, migration experiments revealed their capability to impede LMWH‐induced cell wound healing.

Exogenously heparin from liver cells influences the proliferation of colon cancer cells by increasing the expression of Erb‐B family tyrosine kinase receptors (Fishman et al., 2002; Zvibel et al., 1998). We observed that LMWH led to an increase in the gene and protein expression of ErbB1, ErbB2, and ErbB4, which was effectively suppressed by PEITC, BITC, and SFN. The Erb‐B receptor tyrosine kinase family consists of four cell surface receptors—ErbB1/EGFR/HER1, ErbB2/HER2, ErbB3/HER3, and ErbB4/HER4—that collectively regulate apoptosis, cell motility, proliferation, migration, and differentiation (Habeeb et al., 2023; Holbro & Hynes, 2004). Numerous studies have indicated that alterations in the ErbB receptor are linked to various cancers, with EGFR directly regulating phosphatidylinositol 3‐kinase/protein kinase B (PI3K/Akt) (Androutsopoulos et al., 2023; Ding et al., 2021). In tumor cells, persistent activation of ErbB2 and EGFR triggers similar intracellular signaling proteins and pathways as their wild‐type counterparts, including the mitogen‐activated protein kinase (MAPK), PI3K/Akt, and mammalian target of rapamycin (mTOR) pathways, Src kinase, and signal transducer and activator of transcription (STAT) factors. Given the significance of ErbB receptors in human cancers, inhibitors targeting ErbB have been approved for cancer treatment (Hassan & Seno, 2022), such as Cetuximab (Erbitux), which targets EGFR and is used to treat advanced colorectal cancer (Hynes & MacDonald, 2009). Considering the reported EGFR overexpression in 25% to 82% of colorectal cancer cells, it is implied from this evidence and our findings that PEITC, BITC, and SFN might hold promise in cancer treatment by inhibiting the expression of Erb‐B family factors. This suggests a potential therapeutic avenue.

Like ErbB2, TGF‐*β* plays a crucial role in cancer development by interacting with ErbB2 to promote tumor progression. Some studies suggest that targeting the ErbB2/TGF‐β signaling pathway could have a more effective therapeutic effect (Yingling et al., 2004). This signaling pathway plays a pivotal role in regulating cell proliferation, differentiation, apoptosis, adhesion, invasion, and the cellular microenvironment. Consequently, the malfunction of this pathway can lead to adverse outcomes, and significant changes in its composition are imperative for the processes of carcinogenesis and cancer progression (Meulmeester & Ten Dijke, 2011). TGF‐*β*, which is commonly expressed in the advanced stages of colorectal malignancy, enhances the production of carcinogenic growth factors (Habeeb et al., 2023). It also integrates signals from ErbB receptors and integrins, crucial for promoting cancer cell migration and survival (Wang et al., 2009). Our study indicates a notable increase in TGF‐*β* expression in response to LMWH treatment, and BITC demonstrates a significant ability to counteract this trend. Furthermore, in the apoptotic cell death pathway, Bax promotes cell apoptosis, while Bcl‐2 impedes Bax‐mediated cell apoptosis (Zhang et al., 2022). ITCs possess the capability to counteract heparin's suppression of Bax and enhancement of Bcl‐2, implying that ITCs may exert an anticancer effect by fostering the apoptosis of cancer cells.

## 5 CONCLUSION

The findings from this study both corroborate existing knowledge and introduce new insights regarding the interactions between isothiocyanates (ITCs) and low‐molecular‐weight heparin (LMWH) in human cancer cells, particularly in the areas of cell proliferation and migration, wound healing, apoptosis, and growth factor expressions. Among the four ITCs studied, PEITC, BITC, and SFN exhibited the most significant anticarcinogenic effect against LMWH‐induced cell proliferation and migration. These ITCs appear to exert their effects primarily through the downregulation of Erb‐B family proteins and mRNA expressions, TGF‐*β*, and regulatory apoptotic factors. While various studies have focused on the role of ITCs in cancer research, this is the first examination of their impact on LMWH‐stimulated colon cancer cells. The findings highlight the potential of ITCs, whether as part of a diet or as small molecule drug or supplement, to counteract the negative side effects of exogenous LMWH‐induced colon cancer cell proliferation. This could potentially extend survival and improve the quality of life for cancer patients.

## AUTHOR CONTRIBUTIONS

**Yizi Zhang:** Conceptualization (lead); data curation (lead); writing – original draft (lead). **Karabi Saha:** Methodology (supporting). **Raj Nandani:** Methodology (supporting). **Jiahui Yuan:** Data curation (equal); writing – review and editing (equal). **Moul Dey:** Data curation (supporting); supervision (equal); writing – review and editing (equal). **Zhengrong Gu:** Data curation (equal); supervision (equal); writing – review and editing (equal).

## ACKNOWLEDGMENTS

This research was funded by grants from the South Dakota Biofilm Science and Engineering Center (NSF‐EpsCor Track‐1, No: SA2000397).

## CONFLICT OF INTEREST STATEMENT

The authors declare that they have no known competing financial interests or personal relationships that could have appeared to influence the work reported in this paper.

## Supporting information


Table S1.

## Data Availability

The data that support the findings of this study are available on request from the corresponding author.
