# Supplementary material for: Isothiocyanates attenuate heparin‐induced proliferation of colon cancer cells in vitro
Source: Food Sci Nutr. 2024 Aug 13;12(10):7842–53. doi: 10.1002/fsn3.4296 (PMC11521738; doi:10.1002/fsn3.4296)
Supplement: Supplementary file 1 — Table S1. [file FSN3-12-7842-s001.docx]

Table S1. Oligonucleotide primers used for qRT-PCR

| Gene | Forward | Reverse |
| --- | --- | --- |
| GAPDH | GGACCTGACCTGCCGTCTAG | GTAGCCCAGGATGCCCTTGA |
| TGF-β | ACCTCGGCTGGAAGTGG | CCGGGTTATGCTGGTTGT |
| ErbB1 | GGGTGCAGGAGAGGAGAA | CTGGTTGTGGCAGCAGTC |
| ErbB2 | CGTGGAGAACCCCGAGTA | TGAAGGTGCTGGGTGGA |
| ErbB3 | GAGTGGACGGCAGAGTTTT | TCCAGCAGAGAACCCAGA |
| ErbB4 | CTGAGTTTTCAAGGATGGCT | TGGAAGTATAGATGGGAGGTG |
| Bcl-2 | GCGGATTGACATTTCTGTG | CATAAGGCAACGATCCCA |
| Bax | ATGGGCTGGACATTGGAC | GGGACATCAGTCGCTTCAG |
